# Supplementary material for: Justification of research using systematic reviews continues to be inconsistent in clinical health science—A systematic review and meta-analysis of meta-research studies
Source: PLoS One. 2022 Oct 31;17(10):e0276955. doi: 10.1371/journal.pone.0276955 (PMC9621455; doi:10.1371/journal.pone.0276955)
Supplement: S1 Protocol — (PDF) [file pone.0276955.s002.pdf]

1. ***Review title.***
   The use of systematic reviews when justifying new clinical health studies – a protocol for a systematic review

2. ***Original language title.***
   (not applicable)

3. ***Anticipated or actual start date.***
   01 March 2019 (search)

4. ***Anticipated completion date.***
   01 March 2021

5. ***Stage of review at time of this submission.***
   Preliminary searches ticked as started - pilot of the study selection process ticked as started – considerations of ROB guideline ticked as started.

6. ***Named contact.***
   Associate professor Jane Andreasen

7. ***Named contact email.***
   jaan@rn.dk

8. ***Named contact address***
   DK – Hobrovej 18-22, 9000 Aalborg

9. ***Named contact phone number.***
   +45 22199515

10. ***Organisational affiliation of the review.***
    Department of Health, Science and technology, Aalborg University in collaboration with The Evidence-Based Research Network

11. ***Review team members and their organisational affiliations.***
    1. *Associated Professor Birgitte Nørgaard, University of Southern Denmark*
    2. *Associated Professor Carsten Bogh Juhl, Research Unit for musculoskeletal function and physiotherapy, University of Southern Denmark, and Department of Physiotherapy and Occupational Therapy, University Hospital of Copenhagen, Herlev and Gentofte, Denmark*
    3. *Associate Professor Eva Draborg, DaCHE - Danish Centre for Health Economics, Department of Public Health, SDU*
    4. *Associate Professor Jennifer Yost, M. Louise Fitzpatrick College of Nursing, Villanova University, USA*
    5. *Klara Brunnhuber, Digital Content Services, Elsevier, London, UK*
    6. *Dr. Karen Robinson, Johns Hopkins University School of Medicine, Baltimore, USA*
    7. *Professors, Hans Lund, Centre for Evidence-Based Practice, Western Norway University of Applied Sciences, Bergen, Norway*

12. ***Funding sources/sponsors.***
    No funding

13. ***Conflicts of interest.***
    No conflicts of interest

14. ***Collaborators.***
15. ***Review question.***
    The aim of this systematic review is to identify and synthesis results from primary studies evaluating if and how authors of clinical studies use systematic reviews to justify their study.

16. ***Searches.***
    We will search the following electronic bibliographic databases: MEDLINE (OVID), Embase (OVID), Cochrane Methodology Register. Furthermore, the reference lists of included studies will be checked to identify additional articles and experts' own literature libraries will be screened.
    Abstracts from the last five years of Cochrane Colloquium will be screened.

    Title / Abstract screening *Title / abstract from searches in electronic databases will be screened by two independent reviewers (JA & BN). If one reviewer is in doubt about inclusion or exclusion the reference will be reviewed in full text.*

    Full-Text screening
    Two independent reviewers (JA & BN) will screen the full text. Agreement will be achieved by discussion.

    Prior to importation to Rayyan duplicates will be removed in Endnote.

17. ***URL to search strategy.***

18. ***Condition or domain being studied.***
    The use of systematic reviews in the justifying new clinical studies

19. ***Participants/population.***
    Not applicable

20. ***Intervention(s), exposure(s).***
    Not applicable

21. ***Comparator(s)/control.***
    Not applicable

22. ***Types of study to be included.***
    We will include meta-research studies (or studies performing research on research)

23. ***Context.***
    Clinical health science.

24. ***Primary outcome(s).***

Percentage of primary studies using systematic reviews to justify their study

Qualitative text analysis of how systematic reviews was used in the text to justify the study

Percentage of studies referring to relevant systematic review(s)

25. ***Secondary outcome(s).***

Not applicable

26. ***Data extraction (selection and coding).***

When all references have been screened following the electronical database search, the reference lists of all included studies will be screened by two independent reviewers (JA & BN).
Further, the proceedings from the last 5 years of Cochrane Colloquiums will be screened, and possible relevant abstracts will be looked up in electronic databases to find a possible later publication. If new studies (compared to the electronic database search) are added the reference lists of these new included studies will be screened too.
All screening will be performed using the free software Rayyan (https://rayyan.qcri.org/welcome).
The following information will be extracted by two independent reviewers (JA & BN) from each of the included studies: Bibliographic information; study aims; study design; comparator (if applicable); setting; country; time of data collection; involved professions; results; conclusion; implications for practice/research; strengths and limitations.

Percentage of primary studies using systematic reviews to justify their study

Qualitative text analysis of how systematic reviews was used in the text to justify the study

Percentage of studies referring to relevant systematic review(s)

27. ***Risk of bias (quality) assessment.***

Risk of Bias in SR of EBR related papers

Internal validity

1. *Clear and focused aim*
2. *Good match between aim and chosen method(s)*
3. *Was potential confounding factors identified and taking into consideration if there was?*
4. *Was the chosen source the best alternative among others? (Selection bias)*
5. *Was the variable(s) chosen (A) the most important, (B) same for all included sources?*
6. *Was important variables neglected?*
7. *Did the data collection relied on subjective evaluation or was it unambiguous data that could be identified explicitly?*
8. *Could the classification of the variables / answers have been affected of a prior knowledge about the results?*
9. *Was an appropriate analysis method chosen?*
10. *Was there missing data?*
11. *Was any possible systematic error or biases taken into consideration in the data collection and/or analysis?*
12. *Conclusion supported by the data*

Reporting quality

1. *Was there a well described and unambiguous aim?*
2. *Was the methods well described?*
3. *Was all relevant results reported?*

4. *Was there a list of included studies (sources) (in text or appendix)?*
5. *If studies (sources) was excluded, was the exclusion acceptable explained?*
6. *Was all phases of the methods transparently described?*

28. ***Strategy for data synthesis***
We assume that this will be a configurative review, but if aggregated data emerge, meta-analysis will be conducted. Even though aggregated data should be accessible, a narrative analysis might be chosen dependent on the heterogeneity of the included studies.

29. ***Analysis of subgroups or subsets.***
No planned subgroup analysis

30. ***Type and method of review.***
The information required here relates to the topic and outcome of the type of studies included.

31. ***Language.***
English

32. ***Country.***
Denmark

33. ***Other registration details.***
EBR Network Road Map number 201753

34. ***Reference and/or URL for published protocol.***
None

35. ***Dissemination plans.***
A paper will be submitted to a leading journal in this field

36. ***Keywords.***
Systematic review; justifying new studies; evidence-based research; EBR

37. ***Details of any existing review of the same topic by the same authors.***

38. ***Any additional information.***
This review is carried out as part of the Evidence-Based Research Network (ebrnetwork.org)
