# Supplementary material for: Justification of research using systematic reviews continues to be inconsistent in clinical health science—A systematic review and meta-analysis of meta-research studies
Source: PLoS One. 2022 Oct 31;17(10):e0276955. doi: 10.1371/journal.pone.0276955 (PMC9621455; doi:10.1371/journal.pone.0276955)
Supplement: Attachment — Submitted filename: Response letter 20092022.docx [file pone.0276955.s006.docx]

**Response letter**

Thank you for the opportunity to revise the manuscript. Thank you to the reviewer for the relevant comments concerning the manuscript. We have revised the manuscript in accordance with these comments by addressing all issues from the editor and from the reviewers below.

6. Review Comments to the Author

Please use the space provided to explain your answers to the questions above. You may also include additional comments for the author, including concerns about dual publication, research ethics, or publication ethics. (Please upload your review as an attachment if it exceeds 20,000 characters)

Reviewer #1: I am happy with the responses and thank the authors for their detailed replies, but just had 2 minor issues

*Response: Thank you very much.*

This probably reflects my ignorance so apologies to the authors, but I still do not understand the relationship between the 95% CI around the pooled percentage, and the 'broad prediction interval' which follows it. Could they add a line to explain?

*Response: We have revised and explained more in detail and hope the revised text explains this more clearly.*

*Changes to text:*

*Where the confidence interval showed the precision of the pooled estimate in a meta-analysis, the prediction interval showed the distribution of the individual studies. The heterogeneity in the meta-analysis assessed by I^2^ was 94%. The clinical interpretation of this large heterogeneity is seen in a the very broad prediction interval ranging from 16 to 71%, meaning that based on these studies there is 95% chance that the results of the next study will show a prevalence between 16 to 71%.*

There are some typos remaining. The phrase 'regardless study type' should read 'regardless of study type'.

*Response: Thank you, we have revised as suggested.*

There are some rogue apostrophes in the tables (SR's, RCT's) which need to be edited

*Response: Thank you for pointing this out. We have edited this now.*

On behalf of the author group,

Jane Andreasen
